# Not so NICE guidelines for patients with aortic aneurysms

**DOI:** 10.1186/s42155-019-0081-1

**Published:** 2019-11-12

**Authors:** Ian Loftus

**Affiliations:** St George’s University Hospitals NHS Foundation Trust, London, UK

Recent draft guidelines from The National Institute for Health and Care Excellence (NICE) regarding the management of patients with abdominal aortic aneurysms have proven to be controversial, leading to a prolonged period of consultation and review (Reekers 2019). The details are available online at https://www.nice.org.uk/guidance/conditions-and-diseases/cardiovascular-conditions/aortic-aneurysms.

NICE was established to reduce variation in the availability and quality of treatments provided by the National Health Service (NHS). While government funded, it represents an independent body. The legislation relating to NICE means that any guidance produced only applies in England.

Guidance are drawn up by independent Guideline Development Groups. Once a specific topic is referred to NICE, the group is selected to include medical professionals with clinical and academic expertise, and representatives of patient and carer groups.

They work to a defined rigorous process of literature analysis, weighted towards randomised controlled trials (Rawlins 2015). This aims to assess value for money by calculating additional ‘Quality Adjusted Life Years’ (QALYs) that each treatment modality confers. Therefore in terms of aortic aneurysms, QALYs are calculated by estimating the number of years each treatment modality provides benefit and any change in quality of life each provides.

The number of extra QALYs is then set against the calculated cost of open (OR) or endovascular repair (EVAR), to get a ‘cost per QALY’ for each. The net health benefit can be then be derived. When combined with the cost of treatment, this can be used to estimate an incremental cost-effectiveness ratio (ICER). In general, NICE accepts an ICER of less than £30,000 per QALY.

Once a guideline is completed, it enters a process of consultation whereby any interested party can provide feedback to NICE. However, the final wording of the guideline remains the remit of the independent committee.

NICE first produced guidelines on EVAR in 2008/9. For this, the early and mid-term outcomes of the 4 randomised controlled trials of EVAR versus OR were analysed in detail. These demonstrated a clear early survival benefit from EVAR, with no difference in mid-term all-cause mortality. There was also a higher reintervention rate for EVAR compared to OR in the mid-term, but a short-term benefit in terms of quality of life.

The NICE guidelines stated that EVAR should be considered as a treatment option for patients undergoing elective aneurysm repair, but not for ruptured aneurysms. NICE reviews guidelines when new evidence emerges or after a significant time has elapsed since publication, and a review process began in 2015, leading to a draft for consultation in 2018.

The draft guidelines published in 2018 presented starkly different recommendations. It should be noted that the guidelines are far reaching, providing guidance on screening, medical management, assessment and diagnosis, as well as perioperative management and surveillance. It is unfortunate that many aspects have been ignored in the controversy surrounding the guidelines relating to the provision of EVAR.

Specifically, NICE state that elective EVAR should not be considered in patients who are deemed fit for OR. Nor should EVAR be considered for patients deemed unfit for OR. Therefore no patient should be offered elective EVAR. However, they have acknowledged that there is no accepted standard of assessment of fitness, and suggested this as an area requiring further research.

Conversely, a recommendation is made that EVAR should be considered in patients with a ruptured aneurysm, largely based on data from the IMPROVE trial (IMPROVE Trial Investigators 2017).

The committee justify these recommendations on the basis of high EVAR maintenance costs and reduced durability, predominantly using further evidence from the four randomised trials. This has been controversial, with many feeling strongly that vascular services have been transformed since the trials. The outcomes in the UK have improved dramatically over the last 10 years, with mortality rates in 2018 from OR and EVAR of 3.2% and 0.7% respectively (Waton et al. 2018).

Concerns have been raised that commissioners of vascular surgery would take the published guidelines at face value and prevent patients from receiving treatment that they would receive if residing in other areas of the Western World. The recommendations are considered to be overtly prescriptive, and clinically unworkable, removing the important element of patient choice. Shared decision making is one off the important recent advantages in modern medicine, which is now completely bypassed by the new draft guideline.

The assessment of “fitness” would be the primary factor determining whether a patient has elective OR or no surgery despite a lack of validated tools. There is always some degree of uncertainty about an individual’s fitness and life expectancy. It seems likely that many patients would be denied treatment and the rate of aneurysm rupture would increase.

Contemporary data from the UK shows that 70% of aneurysm repairs are endovascular. This would be the first that NICE guidance would propose actively withdrawing the predominant surgical treatment for a life-threatening condition.

They would have significant implications for resource utilisation, in particular theatre usage, intensive care and in patient bed provision. It seems unlikely that many centres would be able to provide an endovascular service for ruptured aneurysms without an elective practice.

The draft NICE guidelines are also in conflict with recent international guidelines from the European Society of Vascular Surgery (Wanhainen et al. 2019) and the American Society of Vascular Surgery (Chaikof et al. 2018)). The literature analyses may not be as robust, with less focus on cost effectiveness, but they are considered by many to be more workable.

It is generally accepted that OR should be considered in fitter patients with good life expectancy. Furthermore, in patients deemed very high risk or with limited life expectancy, no intervention should be considered.

EVAR clearly needs to be delivered in more cost-effective way. This could be achieved by shortening hospital stays, reducing re-admission and in particular re-intervention rates, and rationalising surveillance programmes. There should be closer adherence to device ‘Instructions For Use’ to reduce reintervention rates, and national registries should be designed to capture device specific and long term outcomes data.

The draft NICE guidelines should be a wake-up call. It is time to reassess who we treat and how we treat them. It is important that, within a resource limited healthcare system, there is a period of rationalisation of AAA repair. The current debate highlights the shortcomings of the available evidence and further research is now an immediate and high priority.

## Data Availability

Not applicable
